# Comparing Computerised Dietary Analysis with a Ready Reckoner in a Real World Setting: Is Technology an Improvement?

**DOI:** 10.3390/nu9020099

**Published:** 2017-01-31

**Authors:** Jessica Paciepnik, Judi Porter

**Affiliations:** 1 Department of Nutrition, Dietetics & Food, Monash University, Level 1, 264 Ferntree Gully Road, Notting Hill, VIC 3168, Australia; j.paciepnik@hotmail.com; 2 Allied Health Research Office, Eastern Health, 5 Arnold Street, Box Hill, VIC 3128, Australia

**Keywords:** ready reckoner, computerized dietary analysis, nutritional analysis, Bland-Altman

## Abstract

Ready reckoners are used in the clinical setting as a tool for the estimation of nutrient intake. With increasing opportunities for nutrition research, ready reckoners may provide for a more rapid analysis of nutritional intake than computerised methods, often seen as the gold standard for nutritional analysis. This research aimed to determine the level of agreement between ready reckoner and computerised dietary analysis through a secondary analysis of clinical trial data. Participant food intakes were estimated by trained observers using the one-quarter method. Daily energy and protein intake were estimated by the healthcare network ready reckoner and computerised dietary analysis. Agreement between methods was tested using t-tests, correlations and Bland-Altman plots. A correlation between analysis methods was observed (*r* = 0.9086 energy, *r* = 0.8700 protein). Wide limits of agreement were observed for both energy and protein intake. Compared with the computerised method, ready reckoner analysis underestimated energy intake by 600 kJ and protein intake by 5 g. Mean energy and protein intake calculated by each method was significantly different (*p* < 0.0001 energy; *p* < 0.0001 protein). No time differences between analysis methods were observed.  In the clinical setting, practitioners should be aware of the variability of a ready reckoner compared to computerised dietary analysis. Further investigation into the acceptability of ready reckoners as a reliable method of nutrient intake determination, particularly for analysis of nutrition research, is required.

## 1. Introduction

Monitoring patient’s food intake during hospitalisation through the use of simple methods that require minimal training has been identified as an ongoing problem [1]. These food intake data underpin clinical practice since they are used to estimate the nutritional intake of patients for the development of nutrition interventions [2]. However, this process can be difficult and resource-intensive [1]. Nutritional intake may be estimated through the direct analysis of consumed foods or through the use of food composition tables and nutrient databases. While direct analysis methods are considered to be the most accurate, they are often expensive and time consuming [3]. Consequently, a simple and reliable method, such as a ready reckoner, may be preferential [1,4].

Ready reckoners have been defined as “a summary tool that provided average energy and protein contents for each grouped menu item” [2] (p. 762). They are developed across food groups through the averaging of known food compositions. For example, several dessert items may be averaged to produce a single energy and protein value for a single “dessert” item. These averages can then be used to quickly calculate an individual’s intake and have been used in previous nutrition research [1,2,5]. There are conflicting reports as to their accuracy. Researchers have suggested that ready reckoners used in conjunction with food record charts were more likely to have poorer accuracy when estimating energy and protein intake [2]. In contrast, others report that a ready reckoner attached to a visual representation of plate waste allowed for greater accuracy [1].

In order to determine whether a measurement tool, such as a ready reckoner, is accurate, it must be compared to measurements determined by other dietary assessment techniques [4], such as computerised dietary analysis. Computerised dietary analysis is often viewed as the gold-standard for the calculation of nutrient intake with reference databases used as the foundation for analysis. Many computerised dietary assessment programs are in use with food database size varying from 70 to over 23,000 items [6].

The use of ready reckoners during trials may be a cost- and time-effective alternative to the “gold-standard” of computerised dietary analysis. Only one known previous report [2] has explored the level of agreement between these two methods of analysis, which is important within the clinical and research context. This research aimed to investigate the level of agreement between ready reckoner analysis and computerised dietary analysis.

## 2. Materials and Methods 

### 2.1. Study Design

**Figure 1:**
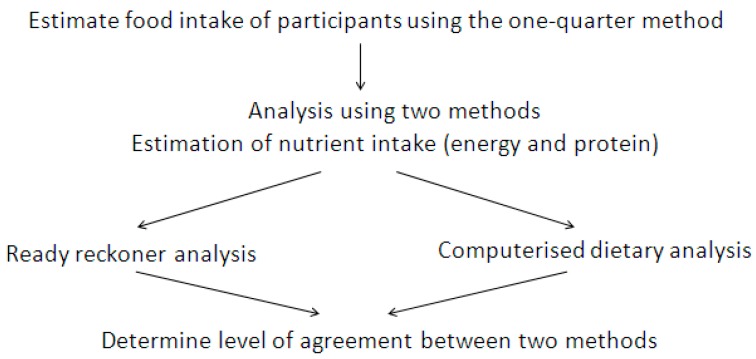
Flowchart of methods used to determine level of agreement in this study.

This was a secondary analysis of data obtained from a clinical trial undertaken in three subacute wards in one large metropolitan Melbourne healthcare network. The clinical trial was registered with the Australian New Zealand Clinical Trials Registry (ANZCTR: 12614001316695); the full trial protocol has previously been published [7]. The flow of methods used to determine level of agreement in this study is illustrated in Figure 1.

The clinical trial from which these data were obtained was conducted under a waiver of consent. As such, patients did not individually provide informed consent. The study was conducted in accordance with the Declaration of Helsinki, and the protocol was approved by the ethics committee of the participating health network (LR69-2014) and the relevant university ethics committee (CF15/414–2015000202).

### 2.2. Estimation of Dietary Intake

Food intake observation of randomly selected participants occurred at main meals (breakfast, lunch and dinner) and mid-meals (morning and afternoon tea). Each main and mid-meal was sighted by observers upon delivery to participants. Food consumption was estimated using the validated one-quarter portion method [8,9]. Intake was estimated using a 5-point visual scale (0, ¼, ½, ¾, all) for each meal component (e.g., cereal, main (meat or vegetarian), starch, vegetables, dessert, drinks, oral nutrition supplement). All observers (3rd year Bachelor of Nutrition and Dietetics students) received intensive training in the use of this technique. Participant self reports were obtained for foods eaten outside the hospital environment.

### 2.3. Ready Reckoner Analysis

The ready reckoner was developed and used by dietitians at the healthcare network hosting the clinical trial. It was designed for use in the clinical setting to estimate a patient’s nutrient intake as part of the nutrition therapy process and had been updated seven months prior to the commencement of the clinical trial when menu changes were made. The ready reckoner included mean values of energy and protein for menu items (e.g., breakfast cereal, soup, meat, meat (wet dishes)). In its development, the nutritional content of items within each meal category were averaged then rounded to the nearest 10kJ energy and 1g protein for each serve size. Values were derived either from product nutrition information panels or from known nutrient composition of each meal as provided by the external production kitchen (unpublished data available at http://www.monashhealth.org/page/nutritional_information), where the majority of meals included in the hospital menu were produced.

Estimates of 24-h food intake of study participants were converted into energy (kJ/day) and protein (g/day) using the ready reckoner. At completion, energy intake was rounded to the nearest 100 kJ and protein intake was rounded to the nearest 1 g.

### 2.4. Computerised Dietary Analysis 

A database containing the nutritional analysis from the commercial production kitchen was integrated into Foodworks (v7.0, Xyris Software ©2012, NUTTAB 2010 database). All other foods consumed were selected directly from the NUTTAB 2010 database. At the completion of the clinical trial, a single research assistant conducted computerised dietary analysis on each 24-h food record using this database. Again, energy intakes were rounded to the nearest 100 kJ and daily protein rounded to the nearest 1 g.

Analysis time for each method was estimated by trained observers for the ready reckoner analysis, and the research assistant who completed the computerised analysis.

### 2.5. Statistical Analysis

Statistical analysis was undertaken using Microsoft Excel 2016 (©2016 Microsoft Corporation). Three participants consumed no food or fluids during the observation period and their records were removed from this analysis. Data were analysed as a whole and within quartiles based on the computerised dietary analysis. Energy and protein intakes were described as mean ± standard deviation and comparisons between analysis methods were made using paired samples t-test and Pearson’s correlation. The Bland-Altman method was used to determine the level of agreement between the two methods. This method calculates the mean difference between two methods of measurement (the “bias”) and 95% limits of agreement as the mean difference (1.96 standard deviation). It is expected that the 95% limits include 95% of the differences between the measurement methods. Bland-Altman plots were used to reveal any relationship between the differences and the magnitude of measurements to look for any systematic bias and to identify possible outliers [10]. The methods were considered to be in agreement if the difference between them was small enough to be considered interchangeable [11]. Authors did not define an a priori limit of variance between methods but rather considered the clinical and research implications of variance where it occurred. Additionally, comparisons of the agreement, under- and over-estimation of the ready reckoner analysis compared with computerised dietary analysis were made.

## 3. Results

The characteristics of study participants who consumed food during the 24-h observation period are shown in Table 1. Due to the nature of the stepped wedge study design, the intake of some participants was recorded on more than one occasion; for the purposes of this analysis all observations have been included. Details regarding unique study participants have been reported elsewhere [12].

Energy intake ranged from 0.2 to 14.8 MJ determined by computerised dietary analysis while a range of 0.3–15.0 MJ was determined by ready reckoner analysis. Protein intake ranged from 3 to 126 g (computerised analysis) and 2 to 139 g (ready reckoner analysis). For both energy and protein intake, the ready reckoner tended to underestimate intake compared to the computerised dietary analysis, with the exception of the first quartile of energy intake where both methods had comparable means and standard deviations (Table 2).

Compared with computerised dietary analysis, the ready reckoner under-estimated 290 (70.2%), over-estimated 103 (25.0%) and obtained agreement for 20 (4.8%) energy intake data pairs. Of the protein intake analyses, the ready reckoner under-estimated 276 (66.8%), over-estimated 135 (32.7%) and obtained agreement for two (0.5%) data pairs.

**Figure 2:**
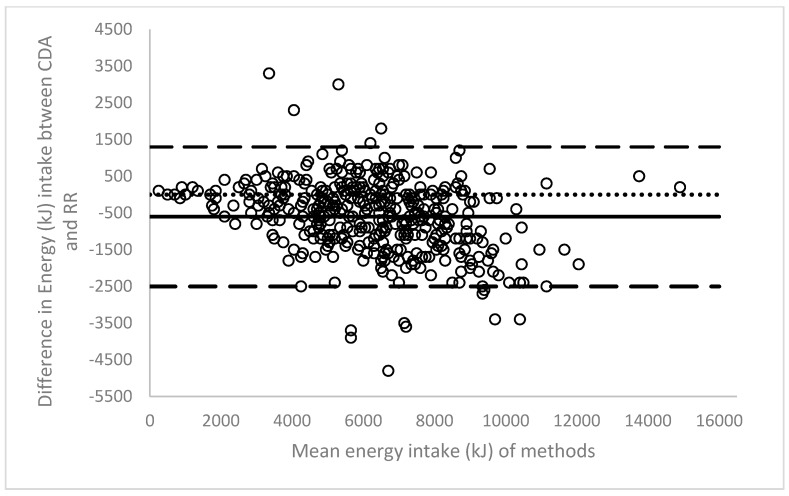
Bland-Altman plot of energy intake calculated using computerised dietary analysis (CDA) and ready reckoner (RR) analysis (*n* = 413).

The line of bias within the Bland-Altman plot indicates an underestimation of 600kJ by ready reckoner analysis across the energy intake dataset (Figure 2). A wide limit of agreement was observed, from −2500 kJ to 1300 kJ.

**Figure 3:**
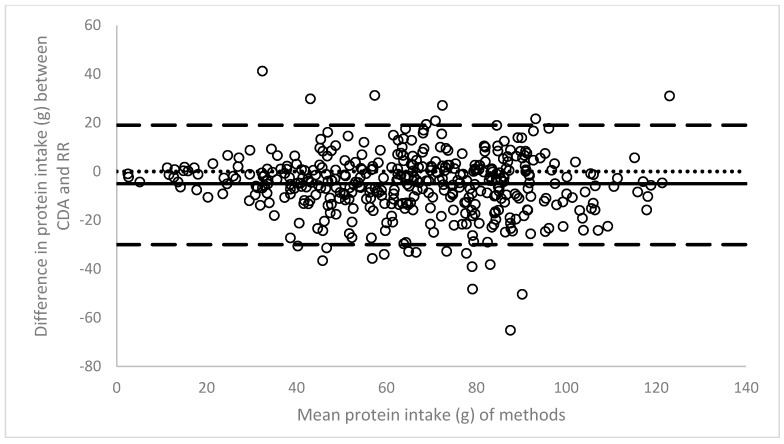
Bland-Altman plot of protein intake calculated using computerised dietary analysis (CDA) and ready reckoner (RR) analysis (*n* = 413).

The line of bias revealed a gap of 5 g between the two methods, again with an underestimation by the ready reckoner method (Figure 3). A trend towards closer agreement was identified towards the lower end of the first protein intake quartile. Overall limits of agreement were wide (−30 g to 19 g).

Trained observers estimated that ready reckoner analysis took 8–10 min per intake record. The research assistant conducting the computerised dietary analysis took 9.8 min to analyse each record.

## 4. Discussion

This study compared ready reckoner and computerised dietary analysis methods of a singular data set, extending the evidence base for practitioners in clinical practice and researchers. Although no time advantages were observed between the techniques, in practice where access to computerised dietary analysis may be a considerable cost or distance, ready reckoner analysis evidently has merits. Equally, however, in the research setting where dietitians and nutrition scientists are conducting clinical trials to inform the development of clinical guidelines and food policy, the underestimation by ready reckoner analysis that we have identified may have implications for reporting and interpretation of results.

The energy and protein intakes reported in this clinical trial were higher than those reported in the only other study of Protected Mealtimes where 24-h intakes were estimated [14]. In the previous research, intakes of (mean ± S.D.) 5011 ± 1774 kJ in the usual care group and 4957 ± 2237 kJ in intervention group were reported. Protein intakes were also lower, with 47 ± 19 g (usual care) and 43 ± 21 g (intervention) reported [14].

In the present analysis a trend of closer agreement was observed within the first quartiles for both energy and protein. This supports a previous report that a ready reckoner was more likely to accurately calculate at smaller intakes and at mid-meals [2]. It is likely that at mid-meals, estimation of consumption is easier to standardise due to smaller intakes and the use of branded goods (such as pre-packaged biscuits). Unlike the study of Palmer et al. [2] reporting generally poor agreement between the two methods tested, this study found wide levels of agreement but high correlation between the two analysis methods. This difference in findings can be attributed to multiple factors, including variation in the interventions being tested and inter-rater reliability.

Measurement error has been defined as “a deviation from true value” [15]. Errors inevitably occur in the determination of food composition and subsequently nutrient intake [16]. It has been suggested that errors in completing intake analysis correctly may result in inaccuracies, most commonly overestimations of greater than 15% [17,18]. The Bland-Altman method allows for the comparison of two methods to determine their level of agreement and takes into account the random error associated with both methods [19]. It acknowledges that the reference method is not infallible and therefore is not truly the “gold standard” [19]. The Bland-Altman analysis does not define whether these limits are clinically acceptable [20], nor does it determine statistical significance.

We suggest that the main cause of difference occurred not from the estimation of food intake (since both were analysed from identical records), but rather from inaccuracies in the development of the ready reckoner itself. It is therefore important to consider the context in which the ready reckoner may be used. This underestimation by the ready reckoner may limit the observation of clinically and statistically significant results when used for research. However, if it is known that a ready reckoner consistently under or over estimates elements of intake, appropriate adjustments may be made at the practitioner level. Therefore, it may be a useful tool to allow for rapid calculation of nutrient intake, particularly in clinical practice.

An advantage of ready reckoners is that they may be utilised rapidly with minimal training [1]. A disadvantage is the averaging of nutrients from multiple samples or recipes during their creation, allowing for the potential accumulation of intrinsic error to occur [4]. Although this was not a validation study whereby authors planned to validate a ready reckoner against computerised dietary analysis, subsequent to this analysis modifications should be made to the ready reckoner to increase the level of agreement between the two methods. Such changes could include more discretion within each descriptor of the menu category—for example specifying “cream-based soup” or “tomato-based soup” rather than “soup”.

There are several limitations within this analysis that should be highlighted. Firstly, the use of the one-quarter method may introduce systematic error in the estimation of food intake. We anticipate that this has not affected the results of either analysis presented here since they were both analysed from the identical dataset. Furthermore, the inter-rater reliability of observers in the use of the ready reckoner was not formally measured although all observers received one full day of training in the study protocol (including estimation of food intake and use of the ready reckoner) prior to the commencement of data collection. This study has only compared the ready reckoner of one health network to computerised dietary analysis and therefore the findings presented may not be representative of all ready reckoners.

## 5. Conclusions

Error in the estimation of nutrient intake is inevitable. However, minimising these errors where possible is vital to allow for the most accurate approximation of intake. While this analysis showed wide limits of agreement between ready reckoners and computerised dietary analysis, there is merit in further investigation to improve the accuracy of the ready reckoner method. Given the error already introduced through estimation of dietary intake, we suggest that computerised analysis should remain the preferred technique for analysis of nutrition research. Clinicians using ready reckoners in practice should be aware of the level of agreement of these methods and account for potential variations in estimating nutritional intake.

## Figures and Tables

**Table 1:** Characteristics of participants who consumed food during the 24-h observation (*n* = 413).

Demographic Variable	Result
Age (years), mean ± S.D.	79.3 ± 12.0
Gender, male, *n* (%)	131 (31.7)
Length of stay (days), mean ± S.D.	57.7 ± 42.1
Body mass index (kg/m^2^), mean ± S.D. (*n* = 410)	24.9 ± 6.3
Subjective global assessment (SGA) ^1^, *n* (%)	*n* = 405
SGA A—well nourished	206 (50.9)
SGA B—at risk or moderately malnourished	184 (45.4)
SGA C—severely malnourished	15 (3.7)
Diet type, *n* (%)	
Full ward diet (including diabetes)	349 (84.5)
Soft diet	36 (8.7)
Other texture modified	7 (1.7)
Other therapeutic diet	21 (5.1)

^1^ Assessed using the Detsky et al. method [13].

**Table 2:** Comparison of 24-h energy and protein intake calculated by computerised dietary analysis and ready reckoner ^1^ (*n* = 413).

Nutrient	Computerised Dietary Analysis	Ready Reckoner Analysis	*r*
Energy (kJ), mean ± S.D.*	6601 ± 2346	5987 ± 2039	0.9086
First quartile (200–5000 kJ)	3500 ± 1200	3600 ± 1300	
Second quartile (5100–6400 kJ)	5700 ± 400	5400 ± 900	
Third quartile (6500–8100 kJ)	7300 ± 500	6600 ± 900	
Fourth quartile (8200–14,800 kJ)	9500 ± 1300	8200 ± 1400	
Protein (g), mean ± S.D.*	68.3 ± 25.0	62.9 ± 24.0	0.8700
First quartile (3–50 g)	35 ± 12	34 ± 14	
Second quartile (51–68 g)	60 ± 5	56 ± 13	
Third quartile (69–85 g)	77 ± 5	73 ± 13	
Fourth quartile (86–126 g)	98 ± 11	87 ± 15	

^1^ quartiles based on computerised dietary analysis; * mean intake compared using paired samples *t*-test, mean intake significantly different *p* < 0.0001.

## References

[1] Budiningsari D., Shahar S., Manaf Z.A., Susetyowati S. (2016). A simple dietary assessment tool to monitor food intake of hospitalized adult patients. J. Multidiscip. Healthcare.

[2] Palmer M., Miller K., Noble S. (2015). The accuracy of food intake charts completed by nursing staff as part of usual care when no additional training in completing intake tools is provided. Clin. Nutr..

[3] West C.E., van Staveren W.A., Maretts B.M., Nelson M. (1997). Food consumption, nutrient intake, and the use of food composition tables. Design Concepts in Nutritional Epidemiology.

[4] Nelson M., Margetts B.M., Nelson M. (1997). The validation of dietary assessment. Design Concepts in Nutritional Epidemiology.

[5] Palmer M., Huxtable S. (2015). Aspects of protected mealtimes are associated with improved mealtime energy and protein intakes in hospitalized adult patients on medical and surgical wards over 2 years. Eur. J. Clin. Nutr..

[6] Probst Y.C., Tapsell L.C. (2005). Overview of Computerized Dietary Assessment Programs for Research and Practice in Nutrition Education. J. Nutr. Educ. Beh..

[7] Porter J., Haines T., Truby H. (2016). Implementation of protected mealtimes in the subacute setting: Stepped wedge cluster trial protocol. J. Adv. Nurs..

[8] Berrut G., Favreau A.M., Dizo E., Tharreau B., Poupin C., Gueringuili M., Fressinaud P., Ritz P. (2002). Estimation of calorie and protein intake in aged patients: Validation of a method based on meal portions consumed. J. Gerontol. A Biol. Sci. Med. Sci..

[9] LaChance P.A. (1976). Simple research techniques for school foodservice. II. Measuring plate waste. Sch. Foodserv. J..

[10] Bland J.M., Altman D.G. (1986). Statistical methods for assessing agreement between two methods of clinical measurement. Lancet.

[11] Portney L.G., Watkins M.P. (2009). Foundations of Clinical Research: Applications to Practice.

[12] Porter J., Haines T., Truby H. (2017). The efficacy of Protected Mealtimes in hospitalised patients: A stepped wedge cluster randomised controlled trial. BMC Med..

[13] Detsky A., McLaughlin J., Baker J., Johnston N., Whittaker S., Mendelson R., Jeejeebhoy K. (1987). What is subjective global assessment of nutritional status?. J. Parenter. Enter. Nutr..

[14] Young A.M., Mudge A.M., Banks M.D., Ross L.J., Daniels L. (2013). Encouraging, assisting and time to EAT: Improved nutritional intake for older medical patients receiving protected mealtimes and/or additional nursing feeding assistance. Clin. Nutr..

[15] Lovegrove J.A., Hodson L., Sharma S., Lanham-New S.A. (2015). Nutrition Research Methodologies.

[16] Gibson R.S. (2005). Principles of Nutritional Assessment.

[17] Simmons S.F., Reuben D. (2000). Nutritional intake monitoring for nursing home residents: A comparison of staff documentation, direct observation, and photography methods. J. Am. Geriatr. Soc..

[18] Pokrywka H.S., Koffler K.H., Remsburg R., Bennett R.G., Roth J., Tayback M., Wright J. (1997). Accuracy of patient care staff in estimating and documenting meal intake of nursing home residents. J. Am. Geriatr. Soc..

[19] Earthman C.P. (2015). Body composition tools for assessment of adult malnutrition at the bedside: A tutorial on research considerations and clinical applications. JPEN.

[20] Giavarina D. (2015). Understanding Bland Altman analysis. Biochem. Medica.

